# The JDCS Model and Blue-Collar Bullying: Decent Working Conditions for a Healthy Environment

**DOI:** 10.3390/ijerph16183411

**Published:** 2019-09-14

**Authors:** Georgia Libera Finstad, Antonio Ariza-Montes, Gabriele Giorgi, Luigi Isaia Lecca, Giulio Arcangeli, Nicola Mucci

**Affiliations:** 1 Business@Health Laboratory, Via degli Aldobrandeschi, 190, 00163 Rome, Italy; g.liberafinstad@gmail.com; 2 Management Department, Universidad Loyola Andalucía, 14004 Cordoba, Spain; ariza@uloyola.es; 3 Department of Business Administration, Universidad Autónoma de Chile, Santiago 7500912, Chile; 4 Department of Human Sciences, European University of Rome Via degli Aldobrandeschi, 190, 00163 Rome, Italy; gabriele.giorgi@unier.it; 5 Department of Experimental and Clinical Medicine, University of Florence Largo Piero Palagi 1, 50139 Florence, Italy; giulio.arcangeli@unifi.it (G.A.); nicola.mucci@unifi.it (N.M.)

**Keywords:** work-related stress, blue-collars, job strain, workplace bullying, psychological health

## Abstract

Violence in the workplace and its health consequences still represent one of the main obstacles to obtaining decent working conditions. In particular, blue-collar workers run a greater risk of experiencing episodes of violence, also because of a lack of autonomy and fewer social interactions. According to the work environment hypothesis, factors such as high workload, lack of social support and lack of job control represent the antecedents of workplace bullying. Following the job demand-control-support model (JDCS), violence can be the symptom of a high-strain environment. Moreover, it is still unclear if workplace bullying can mediate the effects of work-related stress on workers’ health outcomes. The aim of the present study is to analyse the relationship between the components of the JDCS and the health of the workers considering workplace bullying as a mediating variable. By a cross sectional study design, we tested the following theoretical hypotheses: first, JDCS components (conceptualized as stress) are supposed to significantly predict the level of workers’ health. Second, workplace bullying is supposed to mediate the relationship between the JDCS components and the level of health. The sample consists of 400 blue-collars from three different Italian companies. Work-related stress, health outcomes and workplace bullying were measured by specific self-administered questionnaires and the relationships between the variables of interest were tested through a structural equation model (SEM) analysis. The results showed that while the direct relationship between the components of the JDCS and the level of psychological health is weaker (standardized path coefficients SPC = 0.21), the partial mediation hypothesis shows that workplace bullying mediate the relationship between JDCS components and health outcomes (χ^2^/df ratio = 2.70; path from stress to workplace bullying SPC = 0.78; path from workplace bullying to general health SPC = 0.51; *p* = 0.01). The JDCS components (workload, lack of control, lack of support) are useful predictors for workplace bullying. On the other hand, bullying plays a mediating role between the stress experienced and the health consequences. The present study adds new insights into the relationship between violence seen as a form of social behavioural strain and the psychological health of workers. The theoretical and practical implications are discussed. Future research on blue-collars could use longitudinal designs in order to analyse the relationship between social environment, job design and strain reactions.

## 1. Introduction

In a climate characterized by continuous social, political and technological evolutions, working conditions are one of the topics of greatest concern. Job quality is also central to the concept of decent work introduced by the International Labour Organization (ILO). This term refers to the right of the worker to have working conditions that ensure health, safety and dignity [1]. Job opportunities and decent working conditions are also included in the United Nations 2030 Agenda for Sustainable Development. Objective 8.8 of the Agenda underlines the need to ’protect labour rights and promote safe and secure working environments for all workers’ (e.g., absence of physical, mental, or emotional abuse). As outlined by the European Foundation for the Improvement of Living and Working Conditions (Eurofound) one of the most important psychosocial risks at work is the problem of violence and harassment [2]. At European level up to 12% of workers are subject to bullying, verbal abuse, humiliation and sexual harassment [3]. Despite numerous improvements, the emergence of workplace violence is yet to be eradicated. Furthermore, the negative consequences of violence concern both the health of the individuals and the economy of the companies and states [4,5,6,7]. To promote decent work, it is therefore necessary to create a safe work environment. Endeavours should be directed toward the analysis of available data, the development of possible interventions and training programs for managers and supervisors, bearing in mind that the key lies in correct information [8].

The dominant line of research regarding the antecedents of violence concerns the role of the environmental conditions. The work environment hypothesis traces two macro-categories of possible predictors, namely job design (e.g., high workload, lack of skill utilization) and the social environment (e.g., lack of social support). [9,10,11]. Starting from the concepts proposed by the work environment hypothesis several bullying models have been developed. However, these models appear to be too complex or generic, they are difficult to test empirically and often do not provide an adequate list of possible antecedents [12,13].

The job demand-control-(support) model (JDCS) [14,15] is configured as an easily testable model focused on the work environment [16]. According to this model, the worst situation for the workers’ well-being is characterized by high job demands, low job control and low social support. The JDCS model has been one of the main stress models since the 1980s and it is supported by numerous empirical evidence [17,18]. It has been used in many fields and to study a wide range of strain reactions, mostly indicators of well-being. [19]. Nevertheless, what seems to be missing in the literature are studies of social behavioural outcomes that signal a response to stress, like for example, workplace bullying [20]. Interestingly, the components of the JDCS model could be superimposable to the antecedents of workplace bullying described by the work environment hypothesis [21]. This would be also in line with the statement of Salin and Hoel [22]:

‘*It has been argued that the problem of bullying comes to the force when a high degree of pressure is present in a work environment that offers individuals little control over their own work. such an interpretation would be in line with Karasek’s Job Demand Control Model of stress*’

(p. 229).

Nonetheless, only recently scholars have applied the JDCS model to the study of workplace bullying, identifying this phenomenon as a form of social strain. This interpretation therefore hypothesizes the existence of a link between stressful working conditions and behaviours that can, in turn, affect health [23].

Based on the work environment hypothesis this study aims to analyse the relationship between the components of JDCS conceptualized as stress and the consequences on the health of the workers, using violence in the workplace as a mediating variable in a sample of blue-collars. According to the literature, blue collar workers run a higher risk of experience episodes of violence in the workplace [24]. In fact, among blue-collars the prevalence of bullying victims appears higher [25]. Compared to white collars, this occupational group has less restrictive social rules, a lower level of social interactions, less autonomy and less recognition [26].

Supported by previous scholarship, we used the JDCS model for two reasons. This model provides an easily testable theoretical framework and a list of antecedents consistent with the environmental hypothesis of violence [16] Furthermore, this model allows to study a type of social strain such as violence, in response to high pressure work environments. According to the literature, what seems to be missing in the study of strain reactions is the analysis of social-behavioural outcomes (e.g., violence) [20].

### 1.1. Work Environment Hypothesis

Several theoretical models have been proposed for the analysis of the antecedents of workplace bullying. These studies take into consideration the different characteristics that can be traced to two main lines of separate research: the individual-disposition hypothesis and the work environment hypothesis [9,10,27]. A limitation common to both strands of research refers to the fact that it is difficult to admit being a perpetrator of bullying, as a result, information is almost always based on victims’ reports [12,27,28].

The individual disposition hypothesis states that personality traits or the combination of certain characteristics may increase the risk of performing harassment or to be exposed to mobbing episodes [27,29]. Despite the various efforts in identifying key features that act as predictors, scholars have failed to recognize any general profile, which is why this hypothesis receives less support [30].

Factors that have shown greater predictive power are related to the environment conditions and fall into what is called the work environment hypothesis. This hypothesis received considerable support from empirical evidence and constitutes the main line of research [9,10,11,31]. The work environment hypothesis dates back to the work of Leymann [32] who identified a poor work environment and inadequate leadership practices as determinants of bullying. Numerous studies have shown that the risk of workplace violence is correlated with high workload, role conflict and role ambiguity, cognitive demands, emotional demands, physical demands, time pressure, low autonomy, lack of skill utilization, poor leadership and lack of social support [9,10,11,31,33,34,35]. Violence in the workplace has also been associated with physical characteristics of the environment such as crowdedness, high temperatures and noisy workplaces [36].

In the literature there are therefore numerous evidences about the possible environmental antecedents of workplace violence at the individual, group and organizational level and stressful environments are empirically recognized as a possible source of conflict [37].

### 1.2. The Job Demand-Control-(Support) Model

The JDC model developed by Karasek [14] reflects an attempt to unify two trends of research. One dedicated to the study of possible stressors within the work environment and one focused on what is defined as ‘’decision latitude’’ (i.e., the decision-making freedom available to the worker). This model, also known as the job strain model, postulates that psychological strain is not the result of a single environmental variable, but it is the consequence of the demands of a work situation and the freedom that a worker has in coping with these demands.

Job demands, which are identified as stressors, refer to the task requirements or workload and have been operationalized in terms of time pressure and role conflict. Job control, also called decision latitude, refers to the extent to which an individual can have control over work activities. This construct is subdivided into skill discretion (i.e., the possibility of using specific skills) and decision authority (i.e., the autonomy of an individual in decisions regarding the task, for example timing). The strain hypothesis of the JDC states that the situation in which the worker experiences the greatest stress and the lowest well-being is characterized by high job demands and low job control. The buffer hypothesis of the model asserts that control at work can moderate the negative effects of high job demands on well-being. The model also provides an increase in learning parallel to the reduction of stress, thanks to the decision latitude possessed with respect to the task [14,18,38]. Johnson [15] added a component concerning social support (supervisor and colleagues) creating the job-demand-control-support model (JDCS). Indeed, control is not the only resource available to mitigate the effects of workload. In occupational stress research, many studies testify the importance and beneficial contribution of social support in stress and anxiety reduction [39,40]. Within the JDCS model the strain hypothesis is called iso-strain and states that the worst consequences are observed in a situation characterized by high demands, low control and low social support. The buffer hypothesis states that social support moderates the negative effects of high strain (high job demands and low job control) [15].

The JDC and the JDCS models have often been used as a frame of reference for research in various sectors, especially those particularly at risk of stress due to the nature of the job: healthcare, tourism, teaching, safety, etc. [41,42,43,44,45]. The JDCS model is also a useful framework for sectors that include a large portion of manual labour (blue-collar workers) whose work appears to be at high risk of psychophysical stress [46,47]. Furthermore, these models proved to be very useful for the study of several variables such as job dissatisfaction, burnout, turnover, physical and psychological problems related to work (e.g., cardiovascular problems, sleep problems, depression and anxiety), work–family conflict and bullying at work [13,21,48,49,50].

From this perspective, bullying is seen as a symptom of a high-strain environment [20]. For the targets of violence, the process that would link high levels of stress to workplace bullying could concern dysfunctional coping mechanisms and the reduction of available resources. According to the social interactionist theory, work-related stressors (e.g., those identified by the JDCS model) lead to high stress levels, which in turn would lead the individual to become estranged from the stressful situation, violating social norms and expectations (e.g., decreasing work effort or behaving in an avoiding way). These reactions could lead other individuals to adopt hostile behaviours toward the stressed colleague [20,51,52]. The stressful situation could also act on the resources of the worker, no longer able to resist and react and therefore even more susceptible to becoming a victim [36]. Likewise, Lutgen-Sandvik et al. [53] described the environments that favour violence as a ‘boiler room’ in which there is excessive pressure, while Zapf [54] found that according to bullying targets, high stress is the fourth leading cause of violence.

In line with this and following the work environment hypothesis, the JDCS model could be a useful framework for the analysis of workplace violence [13,21,23]. This is also suggested by the leading experts in the workplace bullying field [55]. Nonetheless, there are still few studies on the issue of violence using the JDCS model as a theoretical frame of reference.

For example, Notelaers et al. [21] found that employees in situations of ‘very high job demands’ were two times more likely to be bullied in comparison with the total probability. Employees in ‘very low job control’ situations were almost four-times more likely to be bullied. In a recent study, Goodboy et al. [56] analysed bullying using the JDCS model and the results showed that job demands, job control and social support were useful predictors for bullying reports. The buffer hypothesis was also confirmed since the analyses showed a three-way interaction. Tuckey et al. [23] found that higher levels of workplace bullying (assessed by both targets and observers) were associated with higher levels of job demands along with low job control and low social support. Baillien et al. [16] used a two-wave design with a 6-month time lag to test the JDC assumptions focusing on the targets and perpetrators. The results showed different patterns: workload and control were independently associated with being a victim of bullying while for perpetrators the dimension of workload and control had effects only if they interacted. Eventually, in another study Baillien et al. [13] tested the strain hypothesis of the JDC in matched samples of Spanish and Belgian blue-collar workers. The interaction between the components has predicted the level of victimization showing a stronger association between increased workload and bullying in the low-control condition.

Available data indicate that blue-collars run a higher risk of experiencing episodes of violence. This occupational group has lower levels of autonomy and social support [24,25,26]. These factors have been identified as antecedents of bullying by the work environment hypothesis [9,10,11,31,32,33]. The JDCS model allows to analyse the lack of autonomy and the lack of social support as antecedents of violence seen as a social strain [16,20]. As suggested by the literature, violence can be the result of high-pressure environments (i.e., stressful) and it has devastating effects on the health of individuals. [21,22] Thus, instead of linking job characteristics directly to workers’ health, a connection is sought between working conditions and behaviour patterns (bullying) that can, in turn, influence health [23]. Despite the work environment hypothesis being the dominant line of research, many of the studies concerning workplace bullying remain atheoretical. Nevertheless, workplace bullying is an underrated phenomenon especially in a high-risk category such as blue-collar workers. Moreover, antecedents of workplace bullying, and its health consequences are not totally explained, because of the lack of theoretical framework and data analysis that could confirm theoretical models. Considering these aspects, there is a need to analyse the relationships among predicted antecedents such as work-related stress and organizational factors, and the health outcomes of such workers who experienced work-related stress and bullying reactions.

### 1.3. Aims and Hypotheses

The main objective of the present study is to investigate the links between work-related stress, workplace bullying and health outcomes. Based on the work environment hypothesis and the pre-existing literature this study has a three-fold purpose. First, we aim to investigate the environmental antecedents of workplace bullying through an empirically testable model such as the JDCS.

Second, we use the JDCS model as a theoretical framework considering violence as the consequence of ‘high-strain’ jobs.

Third, using a sample formed exclusively of blue-collars, we aim to add information about this phenomenon in a high-risk occupational group.

A cross-sectional study design was performed to test the following hypotheses:

**Hypothesis** **1:** *High job demands, low social support and low job control will significantly predict the level of health.*

**Hypothesis** **1b:** *High job demands, low job control and low social support will significantly predict reports of workplace bullying, which in turn will predict the level of health.*

We therefore hypothesize a partial mediation model in which stress affects health directly and indirectly by the mediation of workplace bullying.

## 2. Materials and Methods

### 2.1. Participants and Procedure

A study population of 512 workers was recruited over the period 2017/2018 from three different companies: two in the luxury fashion industry (named companies 1 and 2) and one in the construction industry (named company 3). To increase uniformity of the sample, only those subjects who performed a manual job were previously selected and identified according to the contractual framework. These subjects were then invited to participate in the study. For companies 1 and 2 the response rate was 80% (220 and 107 subjects) while for company 3 the response rate was 70% (73 subjects). We thus obtained a sample of 400 workers (51.7% male *N* = 202; 48.3% female *N* = 189). Workers of companies 1 and 2 worked in the areas of production, preparation and cutting of products. Workers of company 3 worked in the areas of plaster processing, maintenance and quarries.

The companies requested support in assessing work-related stress and they organized the administration during working hours considering the time used as paid. Because of the observational nature of the study, and in the absence of any involvement of therapeutic medication, no formal approval of the Institutional Review Board of the local Ethics Committee was required. Nonetheless, all subjects were informed about the study and participation was fully on voluntary basis. The study was conducted in accordance with the Helsinki Declaration.

Each recruited subject completed questionnaires concerning work-related stress assessment, workplace bullying reports and general psychological health, with different modalities based on study group: for companies 1 and 2, the participants had one hour to complete the questionnaire with paper and pencil through a guided administration. Subjects from company 3 completed the questionnaire using an online survey platform called SurveyMonkey (SurveyMonkey Inc.). Participants were informed of the online format through an e-mail inviting participation. The subjects were informed about the objectives of the research and data protection.

Questionnaires were anonymous, coded by the use of barcodes. In the first part of the questionnaire the length of the service and the gender of the respondent were also recorded.

Length of service was classified as a categorical variable, as follow, to ensure anonymity: Length of service <7 years; between 8 and 15 years; >15 years.

Characteristics of selected population are reported in Table 1.

### 2.2. Work-Related Stress Assessment

Job demands, the lack of job control and the lack of social support were measured through the subdimensions of the stress questionnaire (SQ) [57]. The SQ is based on the dimensions of the JDCS model [58], and the UK Health and Safety Executive (HSE) Management Standards Indicator Tool [59]. The statements of the SQ refer to five macro categories: individual, task, physical context, psychological context and socio-economic context. The dimensions of the JDCS model are part of the psychosocial risk scale (individual category), consisting of 25 items in 5 subscales that refer to 5 major psychosocial risks. In this research 4 of the 5 subscales were used (excluding role). Job demands (6 items e.g., ‘my workload is excessive’), lack of job control (5 items e.g., ‘I can plan and schedule the job’ and ‘I can decide when to take a break’), lack of supervisor support (4 items e.g., ‘I can count on the help of the superior/manager when a work problem arises’) and lack of colleagues support (5 items e.g., ‘my colleagues are willing to listen to problems concerning work’). The internal reliability for each subscale, expressed by the Cronbach’s α values, was α = 0.73 job demands, α = 0.67 lack of job control, α = 0.62 lack of supervisor support, α = 0.65 lack of colleague support.

### 2.3. Negative Actions Assessment

The Italian version of the Negative Acts Questionnaire Revised (NAQ-R), validated by Giorgi et al. [60], was used to evaluate the level of negative actions experienced in the workplace. The NAQ-R, previously validated by various studies [61], has a two-factor structure that includes negative behaviours directed to the person or *personal Bullying* (e.g., ‘being ignored or facing a hostile reaction when you approach’) and negative behaviours that refer to the work of the person or *work-related bullying* (e.g., ‘being ordered to work below your level of competence’), consisting of 17 items and 5 items respectively. It contains a Likert scale anchored to specific temporal patterns (5-point Likert-scale ranging from 1 = never to 5 = daily). The score range for each subscale was from 1 to 60 and from 1 to 25 respectively. The internal consistency for each subscale (Cronbach’s α values) was α = 0.91 for personal bullying and α = 0.76 for work-related bullying.

### 2.4. Psychological Health Assessment

The Italian version of the General Health Questionnaire [62,63] GHQ (GHQ-12) was used to assess the level of the psychological health of the participants. The GHQ is used to identify non-specific psychiatric disorders by evaluating the current state of the subject and how this differs from the usual state. Composed of 12 items, it uses a 4-point Likert scale (less than usual, no more than usual, rather more than usual, or much more than usual). The questionnaire is divided into three sub-scales: loss of security (e.g., ‘thinking of self as worthless’), anxiety (e.g., ‘feeling unhappy and depressed’) and social dysfunction (e.g., ‘feeling unable to make decisions’). The score range for subscale was from 0 to 6, from 0 to 12 and from 0 to 28 respectively. The internal reliability for each subscale, expressed by the Cronbach’s α values, was α = 0.75 loss of security, α = 0.81 anxiety and depression, α = 0.79 social dysfunction.

### 2.5. Statistical Analysis

All analyses were performed using statistics software IBM SPSS^®^ (v. 24, package for Windows, SPSS Inc., Chicago, IL, USA), implemented by the AMOS program (Version 23.0 [Computer Program]. Chicago: IBM SPSS). An α-value of 0.05 indicates statistical significance.

Descriptive analyses were performed using parametric statistics, as appropriate. Mean and SD of SQ, NAQ-R and GHQ-12 subscales were calculated for the overall study population, by gender group and by the three study groups. Prevalence rates of categorical variables such as gender and length of service were calculated. The chi-square test was used to test gender differences within the three companies. The one-way ANOVA and Tukey’s post-hoc test were used to compare the means of SQ, NAQ-R and GHQ-12 subscales between the three companies, while t-test compared the same variables between the two genders. Correlations between the variables of interest were calculated using Pearson’s product-moment correlation coefficient with two-tailed test of significance. Finally, the structural equation modelling (SEM) methods were used to test the validity of a partial mediation model, as indicated by hypotheses 1 and 1b in objectives section. To test the goodness of fit achieved by the model the following criteria were considered:

- Relative/normed chi-square (χ2/df*) = values from 2.0 to 5.0 [64] (*df=degree of freedom); root mean square error of approximation—RMSEA = values less than 0.10 [65];
- GFI, AGFI, CFI, IFI = values equal or greater than 0.90 [66].

## 3. Results

In the overall sample, consisting of 400 subjects, males accounted for 51.7% while females were 48.3%. Results of the chi-square test of independence showed significant gender differences in the three companies (*p* < 0.001), with a more pronounced difference detected in company 3 (males 95.9% vs. females 4.1%, Table 1). The mean, standard deviation, minimum and maximum values of SQ, NAQ-R and GHQ-12 are presented in Table 1 (overall values and stratified by company).

For all variables, except for job demands (ANOVA *F* = 8.63, *p* = 0.001), there were no significant differences between companies’ means as determined by one-way ANOVA (Table 1). In detail, job demands were statistically significantly lower in company 2 (2.32 ±0.63) compared to company 1 (2.64 ± 0.71) and company 3 (2.60 ± 0.59) (Tukey post-hoc test *p* < 0.001 and *p* = 0.016, respectively).

No statistically significant results were found between genders for the variables of interest except for work-related bullying and job demands. Work-related bullying was significantly higher in males (8.63 ± 3.60) than females (7.51 ± 11) (t-test *p* = 0.001, not shown in table), as well as detected for job demands (males: 2.64 ± 0.65 vs. females: 2.46 ± 0.69; t-test *p* = 0.011, not shown in table).

The results of the correlations between variables of interest expressed as Pearson’s r coefficients are reported in Table 2. No statistically significant correlation was found between the lack of job control and personal bullying (r = 0.20), social dysfunction (r = 0.13), loss of security (r = 0.16) and anxiety and depression (r = 0.18). Moreover, no statistically significant correlation was found between the lack of colleagues support and social dysfunction (r = 0.16).

The results from the structural equation modelling support the theoretical model, indicating that the partial mediation model fits the data.

The results showed a χ^2^ = 64.90, df = 24. The χ^2^/*df* ratio had a value of 2.70 which indicates a good fit. As hypothesized, the results of the fit indices confirm the goodness of the model. Detailed results for the fit indices are shown in Table 3.

**Figure 1:**
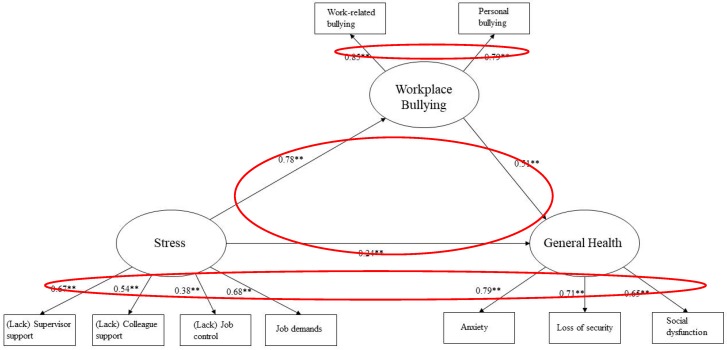
Standardized path coefficients for the partial mediation model (** coefficients are significant at *p* < 0.01).

Examination of the path coefficients for the model showed that the proposed paths were significant (*p* < 0.001) with high standardized values. The model with standardized regression coefficients is shown in Figure 1.

The results show that stress was related to general health directly and indirectly through workplace bullying. The path from stress to general health had a standardized path coefficient (SPC) of 0.24, while the path from stress to workplace bullying had a SPC of 0.78 and the path from workplace bullying to general health had a SPC of 0.51 (*p* = 0.01).

## 4. Discussion

The world of labour, during the past decades, has undergone a period of transitions that has progressively brought attention to the quality of work. Nevertheless, workplace violence still represents an undervalued danger in obtaining decent working conditions [3].

This research, based on the work environment hypothesis, aimed to study how the factors of social environment and job design represent possible antecedents of violence in the workplace. Starting from the characteristics identified by the JDCS model, we have tested a partial mediation model in which violence in the workplace, seen as a form of social behavioural strain, mediates the relationship between these characteristics, identified as ‘stress’, and the health of the worker. The analyses of structural equation modelling confirmed our hypothesis, showing how high job demands together with a lack of autonomy on work activities and a lack of supportive climate lead to workplace violence that, in turn, damage the worker’s health. We also hypothesized that these characteristics would directly influence health without the mediation of workplace bullying. However, as the regression coefficients demonstrate, this relationship is weaker. The results therefore suggest that workplace violence plays a key role in the relationship between the stress experienced by the worker and the consequences on his well-being. Being a target of violence could be seen as the result of a form of social strain. As evidenced by our partial mediation model, decision-making power along with support from colleagues and superiors are important factors for preventing the onset of hostile behaviours. There are in fact some characteristics related to work that, if not properly managed, could create favourable conditions for the occurrence of violence. Work stressors could lead the individual, having no more resources, to exhibit a series of maladaptive behavioural and affective reactions. This in turn could lead colleagues to act with hostility [51,52].

Importantly, the factors identified in this research are in line with previous studies. Indeed, there are many evidences that show how workload, the lack of job control and the lack of support are effective predictors for bullying [10,11,31]. The JDCS model could therefore be used as an etiological explanation for environments that encourage violence [56]. To protect the health of the worker it is essential to control the types of behaviour exhibited in the workplace, avoiding an escalation of conflicts that would damage workers and productivity.

ANOVA results testing differences on stress, bullying and perceived health, did not show significant differences between the different organizations, except for the level of job demands, which was significantly lower in organization 2, compared with the other organizations. Despite the level of job demands was not the same in the three companies, the low variance of the other variables of interest makes the sample sufficiently homogenous, so we considered it as whole.

The values of α Cronbach coefficients showed a good internal consistency for all the subscales of SQ, NAQ-R and GHQ-12 (α > 0.70). Only for the lack of job control, the lack of supervisor support and the lack of colleagues support we found slightly lower values (α = 0.67 lack of job control, α = 0.62 lack of supervisor support, α = 0.65 lack of colleagues support). However, values close to 0.60 are also accepted in the literature [67].

The results of the t-tests showed a higher score for the work-related bullying variable in men than in women. In the literature, there is still no unanimous agreement on the role of gender in workplace bullying. Large-scale studies showed how the incidence of workplace bullying is similar in both men and women [68]. Other studies have shown that exposure to bullying is higher in women, who often represent a minority [10]. Following the social dominance theory, the dominant group uses several forms of oppression to maintain its power [69]. Indeed, the interpretation of workplace bullying is highly anchored to the specific context, as in women-dominated environments, men report a higher level of negative actions experienced [10,70]. Although our blue-collars sample was not dominated by women, men reported a higher level of work-related bullying. A possible explanation of this data could lie in the different coping strategies implemented by women and men. Men are not likely to ask for help and tend to face the bully directly. However, openly addressing the bully may not be an effective strategy and may increase the level of victimization. [71,72]. The different behaviour would find an explanation in the gender role socialization theory, according to which there are different expectations about the role of women and men. Men are typically seen as being able to cope with situations. For this reason, they do not ask for help as a coping resource [73,74].

### 4.1. Limitations and Future Research

This research, despite all the precautions, has some limitations. First, the sample of blue-collars belonged to three different organizations, two in the luxury fashion business and one in the construction business. Despite this diversity, results of stress, bullying and general health levels were quite similar among them, so the risk of bias can be considered as low. Furthermore, our sample was homogeneous and not representative, being formed exclusively of blue-collars. Despite this, de Lange et al. [75], did not find differences between heterogeneous and homogeneous samples in the JDC model study.

Second, the cross-sectional study design does not allow for causal inference about the relationship between stress, bullying and general health. However, a longitudinal research by Baillien et al. [16] showed that job demand and job control precede the onset of workplace bullying rather than following it. Future studies can adopt a longitudinal research design to provide further indications about the causality of the relationship.

Another possible limitation is the exclusive use of self-report measures that could increase the risk of common method variance. However, there is still no unanimous agreement on the magnitude of the effects of the common method variance [76,77]. To reduce the possible influences, all the necessary procedures have been followed, for example, providing participants with information about the anonymity of the answer and the absence of wrong answers [78]. One of the possible solutions lies in the use of multi-method data and the objective measurements. For example, the literature suggests the use of managerial reports or scores from a third party (e.g., researchers). However, there is an ethical problem in the use of scores from a third party, which resides in not intervening when negative behaviours occur in order to collect data [79]. Moreover, the use of an online survey (company 3) increases the risk of possible methodological bias.

Finally, a limitation lies in having used a single model of partial mediation without making a comparison between other models. Only the iso-strain hypothesis of the JDCS model was analysed and not the buffer hypothesis. One of the directions for future research could be to investigate how the variables of control and social support moderate the relationship between high job demands and workplace bullying and how, in turn, workplace bullying mediates the relationship with the health.

Despite those limitations, our findings are in line with the literature. The *‘high-strain job’* situation

has proven to be predictive of the level of workplace bullying. Furthermore, this study represents one of the first attempts to use the JDCS model as a frame of reference for the study of workplace bullying, especially in the field of blue-collars.

### 4.2. Practical Implications

The features of job design and the work environment could be also the starting points for possible interventions. The literature suggests that the factors traced by the work environment hypothesis can also be applied and used in the management field [80]. Consequently, when planning interventions, management should aim to ensure an adequate level of autonomy and a supportive climate to guarantee healthy working conditions [23,81]. A problem related to the blue-collar workers’ professions could be to ensure a commensurate level of autonomy and control despite the repetitive nature of the work. Although the work process is highly standardized, worker empowerment can still be achieved through a control based on interdependence. However, this can only happen if the decision-maker understands that autonomy must be a function of coordination to ensure the system’s integration. The supervisor should be able to observe in a non-judgmental manner in order to allow the employee to have a range of autonomy despite possible errors [82,83]. At management level, one of the practical indications is to increase employees’ sense of initiative [84]. According to Baard [85] an empowering manager guarantees less restrictive rules and offers the possibility of making mistakes as a learning process. The occasions when managers can communicate their support can be, for example, during training sessions or mentoring [86]. Another important factor concerns the possibility of receiving help and therefore being included in a supportive environment. Especially in blue-collars, the belief that superiors are not interested in creating a good work environment represent a stress factor [87]. Blue collar workers tend to establish relationships and evolve around a team or a community. For this occupational group interaction with other people, in addition to being an element of protection against stress is already an element of satisfaction [88]. What is needed to address the issue of blue-collar workers is a shift in perspective. A supervisor who promotes an atmosphere of support and who is able to guarantee an adequate level of autonomy will create an environment that disincentives the display of negative behaviours [56].

Finally, to prevent the occurrence of negative actions, the organization could proceed with the creation of a policy in which it undertakes to obstruct any offensive attitude [89] and the creation of special platforms where employees can communicate their discomfort without the fear of punishment [55].

## 5. Conclusions

The present study adds new insight into the relationship between occupational stress, bullying and general health in blue-collar workers. The theoretical model, tested with the use of structural equation analysis, confirmed the hypothesis that occupational stress, in terms of high job demands, low job control and low social support is related to general health directly and indirectly through workplace bullying.

The contributions of this study concern different aspects. First, the use of an easily testable and specific model constitutes a significant advantage for the field of workplace bullying in which most of the studies remain atheoretical. The use of a stable framework such as the JDCS model can allow the replication of the present study in several work environments. Furthermore, using the JDCS model as a theoretical framework we have extended its use to the bullying domain. As suggested by previous scholars, there is a lack of studies concerning the link between stressful conditions and behavioural outcomes such as violence. Indeed, another contribution of this research lies in the integration of research on job stress and workplace bullying, considering the latter as a form of social strain in response to an excessive stress level experienced by the workers.

Eventually, this research underlines the value of interventions specifically designed for blue-collars. Our results underscore the importance of possible stressors such as lack of decision-making autonomy and an unsupportive environment. Despite the repetitive nature of the work, it is possible to provide a certain degree of job control as an element of defence from stress. Organizations can use this information to create an anti-violence policy and to build specific job designs aimed at increasing workers’ well-being.

Further research focused on blue-collars samples with a longitudinal study design could be useful to fill the lack of studies about social behavioural outcomes that signal a response to stress such as bullying.

## Figures and Tables

**Table 1:** Characteristics of the study population and results of stress questionnaire (SQ), Negative Acts Questionnaire Revised (NAQ-R) and General Health Questionnaire (GHQ-12) stratified by organization and comparisons between organizations.

Variables	Organization 1	Organization 2	Organization 3	Overall sample	Test
	*N* (%)	*N* (%)	*N* (%)	*N* (%)	
Length of employment					
<7 years	165 (75.0%)	90 (84.1%)	8 (11.0%)	263 (65.8%)	
7–15 years	45 (20.5%)	11 (10.3%)	33 (45.2%)	89 (22.2%)	
>15 years	10 (4.0%)	6 (5.6%)	32 (43.8%)	48 (12.0%)	
					Chi-square
Gender					*p* < 0.001
Male	89 (42.0%)	43 (40.6%)	70 (95.9%)	202 (51.7%)	
Female	123 (58.0%)	63 (59.4%)	3 (4.1%)	189 (48.3%)	
	**Mean, SD (min–max)**	**Mean, SD (min–max)**	**Mean, SD (min–max)**	**Mean, SD (min–max)**	**ANOVA**
SQ					
Job demand	2.64 * ± 0.72 (1.00–4.00)	2.32* ± 0.63 (1.00–4.00)	2.61* ± 0.59 (1.00–5.00)	2.55 ± 0.69 (1.00–5.00)	(*F* = 8.63, ***p* = 0.001**)
Lack of job control	2.73 ± 0.76 (1.00–4.00)	2.79 ± 0.60 (1.00–4.00)	2.66 ± 0.68 (1.00–5.00)	2.73 ± 0.70 (1.00–5.00)	(*F* = 0.78, *p* = 0.45)
Lack of social support (colleagues)	2.44 ± 0.69 (1.00–5.00)	2.33 ± 0.68 (1.00–4.00)	2.37 ± 0.63 (1.00–5.00)	2.40 ± 0.68 (1.00–5.00)	(*F* = 1.06, *p* = 0.34)
Lack of social support (superiors)	2.41 ± 0.91 (1.00–5.00)	2.47 ± 0.84 (1.00–5.00)	2.60 ± 0.76 (1.00–5.00)	2.46 ± 0.87 (1.00–5.00)	(*F* = 1.31, *p* = 0.27)
NAQ-R					
Personal bullying	14.94 ± 5.55 (11.00–48.00)	15.36 ± 6.18 (11.00–43.00)	15.26 ± 7.85 (11.00–55.00)	15.11 ± 6.18 (11.00–55.00)	(*F* = 0.18, *p* = 0.82)
Work-related bullying	8.08 ± 3.19 (5.00–24.00)	8.01 ± 3.44 (5.00–22.00)	8.05 ± 3.93 (5.00–25.00)	8.06 ± 3.39 (5.00–25.00)	(*F* = 0.01, *p* = 0.98)
GHQ-12					
Loss of security	0.75 ± 1.31 (.00–6.00)	0.70 ± 1.35 (.00–5.00)	0.44 ± 1.20 (.00–6.00)	0.68 ± 1.30 (.00–6.00)	(*F* = 1.58, *p* = 0.20)
Anxiety	3.82 ± 3.14 (.00–12.00)	3.70 ± 2.97 (.00–12.00)	3.34 ± 2.45 (.00–12.00)	3.70 ± 2.98 (.00–12.00)	(*F* = 0.72, *p* = 0.48)
Social dysfunction	6.07 ± 2.90 (.00–18.00)	5.68 ± 2.54 (.00–16.00)	6.23 ± 2.34 (.00–18.00)	5.99 ± 2.71 (.00–18.00)	(*F* = 1.07, *p* = 0.34)

* Tukey post-hoc test company 2 vs. 1, *p* < 0.001; company 2 vs. 3, *p* = 0.016. Bold: *p* < 0.05.

**Table 2:** Pearson’s correlation coefficients of the variable of interest.

Variable	1	2	3	4	5	6	7	8	9
1. Personal bullying	1								
2. Work-related bullying	0.683 **	1							
3. Social dysfunction	0.386 **	0.404 **	1						
4. Loss of security	0.487 **	0.415 **	0.459 **	1					
5. Anxiety/depression	0.437 **	0.456 **	0.527 **	0.568 **	1				
6. Job demands	0.378 **	0.528 **	0.305 **	0.272 **	0.445 **	1			
7. (Lack) job control	0.196 **	0.221 *	0.129 **	0.158 **	0.184 **	0.209 **	1		
8. (Lack) supervisor support	0.416 **	0.448 **	0.295 **	0.259 **	0.347 **	0.446 **	0.326 **	1	
9. (Lack) colleagues support	0.358 **	0.337 **	0.160 **	0.258 **	0.274 **	0.350 **	0.293 **	0.370 **	1

* = significant at *p* < 0.005; ** = significant at *p* < 0.001.

**Table 3:** Results of fit indices for the partial mediation model.

Model	*x* ^2^	*df*	*x*^2^/*df*	RMSEA	GFI	AGFI	CFI	IFI
Partial mediation model	64.90	24	2.70	0.07	0.97	0.93	0.96	0.97
